# Condensation of Force Field Parameters from Machine Learning Predicted Distributions for High-Throughput Virtual Screening Applications

**DOI:** 10.1021/acs.jcim.5c02199

**Published:** 2025-11-21

**Authors:** Domenico Bonanni, Yuedong Zhang, Davide Gadioli, Gianluca Scarpellini, Pietro Morerio, Alessio Del Bue, Andrea Rosario Beccari, Gianluca Palermo

**Affiliations:** † Department of Physical and Chemical Sciences, University of L’Aquila, 67100 Coppito, Italy; ‡ 121451 Istituto Italiano di Tecnologia, 16163 Genova, Italy; § Dipartimento di Elettronica, Informazione e Bioingegneria, 18981 Politecnico di Milano, 20133 Milano, Italy; ∥ EXSCALATE, Dompé Farmaceutici S.p.A., 80131 Napoli, Italy

## Abstract

Transferable biomolecular force fields are developed by fitting either ab initio or experimental data related to representative molecules and can then be used to model chemical entities that are similar to the ones they were developed for. However, once parametrized on a given dataset, they are difficult to refit once new chemical entities, sensing schemes, or functional forms are introduced. On the other hand, Machine Learning Force Fields (MLFF) have recently gained attention for their accuracy and ease of expanding their Applicability Domain (AD). Nonetheless, their prediction times make them incompatible with High-Throughput Virtual Screening (HTVS) requirements. In this work, we follow the inverse of the widely adopted approach with transferable force fields and propose a new condensation approach that takes advantage of machine learning algorithms to massively predict force field parameters. The generated numerical distributions are then condensed in a single value that captures in a statistical way the chemical variability of the underlying molecules sharing that specific force field parameter and giving rise to the distribution itself, improving 30× computational efficiency with limited reduction in predicted molecular geometries accuracy. When tested on the public release of the OpenFF Industry Benchmark Season 1 v1.1 dataset, the molecular structures optimized by minimizing the Potential Energy Surface built with condensed FF parameters only show a minor decrease in Root Mean Squared Deviation (RMSD) and Torsion Fingerprint Deviations (TFD) performances compared to those obtained using molecule-specific FF parameters predicted at runtime. To give more context, the original MLFF and its condensed version are evaluated with respect to several well-known transferable force fields widely used for biomolecular simulations.

## Graphical abstract

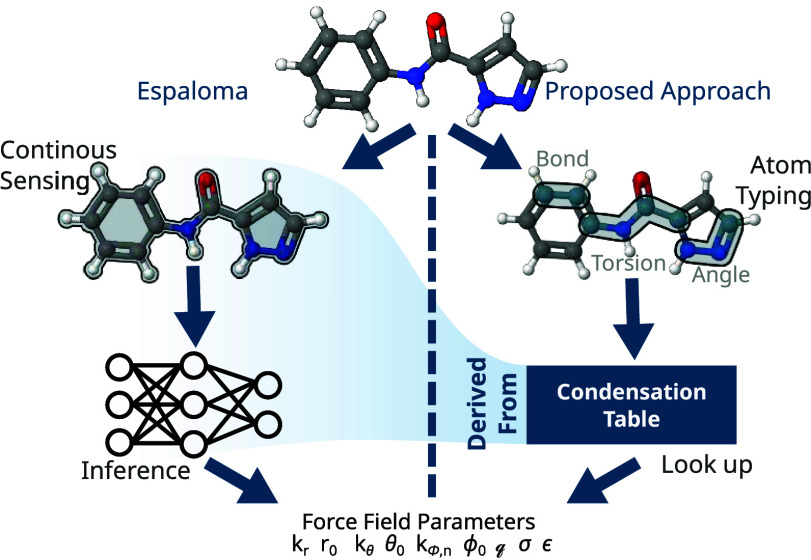

## 1 Introduction

Recent advancements in Geometric Deep Learning (GDL) enabled the development of accurate Machine Learning Force Fields (MLFF) relying upon Graph Neural Networks (GNNs) architectures, with major benefits for computational chemistry and Molecular Dynamics (MD) simulations. In this context, Machine Learning Interatomic Potentials (MLIPs), also called Neural Network Potentials (NNPs), have been widely demonstrated to be capable of predicting energies and forces of small molecules with a comparable accuracy to Quantum Mechanical (QM) methods and orders of magnitude lower computational cost.^1^

Earliest approaches in the development of Neural Network Potentials (NNPs) like ANI,^2^ TensorMol,^3^ AIMNET^4^ were based on Deep Neural Networks (DNN) and used handcrafted descriptors based on local atom-centered convolution functions. Afterwards, to remove the need for predefined convolution functions for deriving atomic descriptors, architectures based on rotationally covariant neural networks like Cormorant^5^ or exploiting Message Passing approaches such as Dimenet,^6^ PAINN,^7^ PAMNET,^8^ SpookyNet,^9^ and TorchMD^10^ were proposed. Recently, state of the art *E*(3) models like NequIP,^11^ MACE,^12^ MACEOFF23,^13^ and Allegro^14^ have gained attention because of their superb prediction accuracy and improved computational cost with respect to legacy ones. Finally, improved predictions of Absolute Binding Free Energies (ABFE) from Molecular Dynamics (MD) simulations were also demonstrated through the integration of Neural Network Potentials and Molecular Mechanics (NNP/MM) methods.^15,16^

Nonetheless, these models are nowadays orders of magnitude more expensive than Molecular Mechanics Force Fields (MMFFs) for tasks such as molecular energy and geometry prediction. This is because MMFFs employ a simple physics-inspired functional form to approximate the QM Potential Energy Surface (PES) of the molecular system at hand, hence, trading accuracy in favor of computational speed. Therefore, they are much more suited to model small molecules in High Throughput Virtual Screening Applications than NNPs and have also become well established enablers of MD simulations of large biomolecular systems up to hundreds of thousands of atoms.^17^ To parametrize a system, transferable MMFFs like Amber^18^ or CgenFF^19^ rely on lookup tables that are populated according to a prespecified set of atom types. Each atom type describes the chemical environment of an atom and is based on properties such as element, hybridization, connectivity, etc. Finally, direct chemical sensing models based on SMIRKS molecular fragments replacing discrete atom types were proposed.^20^ One major benefit of SMIRKS based chemical sensing is the consistent reduction in the number of parameters required to fully specify the force field.^21^

Recently, the Espaloma machine learned force field was demonstrated to achieve comparable performances on the OpenFF Industry Benchmark Season 1 v1.1 ^22^ with respect to established transferable biomolecular force fields such as gaff 2.1.1, OpenFF 2.1.0 and 2.0.0.^23^ Nonetheless, the Espaloma MLFF offers several advantages over the latter, particularly when dealing with functional forms upgrade and force field refitting.^24,25^ These advantages mainly come from the ability of the model to assign valence parameters based on a graph representation of the molecule together with user-defined functional forms, without the need to specify beforehand an explicit chemical sensing model based on atom types. Espaloma employs a graph neural network to construct continuous vectorial representations of atoms based on their chemical environments, inherits non-bonded parameters from OpenFF 2.0.0, and predicts atoms’ electronegativity and hardness to infer AM1-BCC quality partial charges. Finally, it predicts valence parameters through a terminal feed forward neural network stage.

On the same path, Grappa (Graph Attentional Protein Parametrization)^26^ is a recently published open source model that as well inherits non-bonded parameters from OpenFF 2.0.0 but, unlike Espaloma, uses AM1-BCC partial charges as input features, relies on Graph Attentional Neural Network (GANN) to infer local chemical environments and finally exploits symmetric transformers to predict valence parameters. Grappa was recently demonstrated to outperform several established biomolecular force fields, as well as Espaloma itself in predicting energies and forces on the Espaloma test set. However, despite being much faster than NNPs in calculating biomolecular systems’ PES, this molecule parametrization at runtime is still not compatible with High-Throughput Virtual Screening (HTVS) campaign requirements.

The goal of this paper is to propose a novel condensation scheme to calculate statistically validated force field parameters distilled from related numerical distributions massively generated offline by MLFFs on a representative training set. The main benefit of this approach is the removal of the computational burden commonly associated with MLFFs predictions at runtime. Our method allows to capture on a statistical basis the effect different chemical environments have on force field parameters related to specific atom-types. Condensed force field values can then be stored in lookup tables to be quickly retrieved upon parsing of new molecular entities in High-Throughput Virtual Screening (HTVS) campaigns. In this work, the MMFF94 force field chemical sensing scheme has been used.^27^

The paper is structured as follows:

- In Section 2 we describe functional forms underneath Class I and Class II force fields.
- In Section 3.1 we briefly outline Ligen-Pre Software used to generate molecular conformers using Espaloma predicted force field parameters.
- In Section 3.2 we describe data, workflows and integrated software programs used for Espaloma model retrain.
- In Section 3.3 we describe the workflow used for calculating statistically validated Class I bonded parameters.
- In Section 4.1 we benchmark computational performances from Condensed Espaloma model with respect to its original and Class II counterparts.
- In Section 4.2 we assess on the OpenFF Industry Benchmark Season 1 v1.1 dataset original Class I, Class II and condensed Espaloma models’ performances against several well-established biomolecular force fields.
- Finally, in Section 5 we highlight Active Learning strategies to increase Condensed Espaloma model’s Applicability Domain.

## 2 Theory

### 2.1 Espaloma Class I Machine Learned Force Field

In Molecular Mechanics, the potential energy function used to model the interactions between the atoms of the system is called the “force field”, which is made up of analytical functions depending on the internal coordinates of the system and on a number of parameters to be fitted on either ab initio or experimental (e.g., crystallographic, spectroscopic, etc.) data. Each energy term describes a type of interaction that is classified based on the number of atoms involved. Class I force fields employ uncoupled harmonic functions to model the energetic cost of bond stretching, angle bending, and improper torsions, while proper torsion are modeled by means of periodic functions. There are no cross-terms, i.e., each energetic term depends on only a single internal coordinate.

In the Espaloma 0.3.1 model, the following functional forms are employed:

1 $$\{\begin{array}{l} U_{\mathrm{bond}}\left(r;k_{r},r_{0}\right)=\frac{k_{r}}{2}{\left(r-r_{0}\right)}^{2},,\\ U_{\mathrm{angle}}\left(\theta ;k_{\theta },\theta_{0}\right)=\frac{k_{\theta }}{2}{\left(\theta -\theta_{0}\right)}^{2},,\\ U_{\mathrm{torsion}}\left(\phi ;k_{\phi ,n},\phi_{0}\right)=\overset{n_{\max}}{\underset{n=1}{\sum }}k_{\phi ,n}\left[1+\cos n\left(\phi -\phi_{0}\right)\right],,\\ U_{\mathrm{Coulomb}}\left(r;q_{i},q_{j}\right)=\frac{1}{4\pi \epsilon_{0}}\frac{q_{i}q_{j}}{r},,\\ U_{\mathrm{vdW}}\left(r;\sigma ,\epsilon \right)=4\epsilon \left[{\left(\frac{\sigma }{r}\right)}^{12}-{\left(\frac{\sigma }{r}\right)}^{6}\right], \end{array}$$

where variables are listed before semicolon, while parameters are listed after it.

The first term models two-body interactions, i.e., bonds vibration energy. A harmonic potential is used where *r*_0_, which represents the length of the bond corresponding to the minimum energy, and the force constant *k*_*r*_ depends on bond type.

The second term models three-body interactions and accounts for the energy cost due to valence angle deformation (three-body interaction). This also has the form of a harmonic potential, where θ_0_ represents the reference valence angle and *k*_θ_ represents the force constant.

The third term models four-body interactions in the form of proper torsions, i.e., it accounts for single bond torsional energy strain. It uses a sum of trigonometric functions depending on dihedral angles’ values. In such functions, *k*_ϕ,*n*_ is the *n* ^th^ force constant and ϕ the phase.

The fourth and fifth terms describe electrostatic interactions between pairs of non-bonded atoms *i* and *j* with charges *q*_*i*_ and *q*_*j*_ separated by distance *r*_*ij*_.

In particular, the fourth term describes Coulomb interactions. In this work, AM1-BCC quality partial charges are predicted using the Espaloma model, which in turn exploits a simple charge-equilibration scheme that learns electronegativity and hardness parameters following the protocol described in.^25^ In this approach, “electronegativity” $e_{i}=\frac{\partial E}{\partial q_{i}}$ quantifies the desire for an atom to take up negative charge, while “hardness” $s_{i}=\frac{\partial E^{2}}{\partial^{2}q_{i}}$ quantifies the resistance to gaining or losing electronic charge. Atomic charges are then calculated by taking advantage of Lagrange multipliers’ method in minimizing the total electostatic Energy subject to charge constraints as detailed in.^28^ Worth to note, a single value for the relative dielectric constant ϵ is used. Although this approximation is physically unrealistic, the best solution to this problem would be to include atomic polarizability in the potential energy, which would allow in turn to obtain the correct electric field at every point of the simulated system. However, in order to follow this path it would be necessary to introduce the electric field equation which takes into account the presence of dipoles induced on the individual atoms, giving rise to a system of 3N equations in 3N unknowns (each *x*, *y*, *z* component of the atomic positions). Another elegant way of introducing polarizability into the system would be to use point charges localized on each atom and linked together by oscillators of appropriate force constants called Drude oscillators.^29^ Unfortunately, both approaches are scarcely compatible with High-Throughput Virtual Screening speed requirements.

The fifth term accounts for the Lennard-Jones potential, which is one of the most used expressions for modeling van der Waals interactions. It is made up of two terms that respectively, model a short-range repulsive interaction and a long-range attractive one. ϵ and σ are constants that can be obtained by fitting experimental data or by performing quantum mechanical calculations. In the Espaloma 0.3.1 these constants are taken from OpenFF 2.0.0 force field.^24^

### 2.2 Espaloma Class II Machine Learned Force Field

Class I force fields use simple harmonic functional forms for modeling bonded interactions. This approximation works well for small deviations from equilibrium geometries, but it fails to accurately represent molecular behavior under more extreme conditions.^30^ Furthermore, Class I force fields treat bond stretching, angle bending, and dihedral rotations as independent, neglecting the coupling between these internal variables. This approximation can lead to inaccuracies in predicting molecular geometries and properties.^31^

Class II force fields address these limitations by introducing cross-terms (e.g., coupled stretch–bend energy) and anharmonic functional forms (e.g., higher order polynomial bond stretch energy), which are crucial for accurately modeling vibrational frequencies, conformational energies, and torsional energy barriers,.^32,33^ For example, it was shown in ref 31 that the elimination of bond anharmonicity costs 0.4 kcal/mol in the fit of ab initio energies, while neglecting anharmonicity and cross-term interactions causes an increase in the error of calculated forces and curvatures on alkane molecules by factors of 3.3 and 4.0, respectively. Furthemore, RMSE in energy first derivatives, which is an important indicator of force field performance in Molecular Dynamics simulations, more than double. Therefore, Class II force fields allow for more reliable representations of molecular flexibility and conformational changes and are capable of providing more accurate molecular energies and vibrational spectra than their Class I counterparts.^34,35^ Examples of Class II force fields include Consistent Valence Force Field (CVFF),^36^ Extensible and Systematic Force Field,^37^ MM Series, i.e.: MM2,^38^ MM3,^39^ MM4,^40^ and MMFF94.^27^ To assess possible advantages granted by Class II functional forms in molecular structure prediction, the following functional forms were integrated into a separate version of the Espaloma model:

2 $$\{\begin{array}{l} U_{\mathrm{stretch}}\left(r;k_{r},r_{0},cs\right)=\frac{k_{r}}{2}{\left(r-r_{0}\right)}^{2}{\left(1+cs\left(r-r_{0}\right)+\frac{7}{12}cs\left(r-r_{0}\right)\right)}^{2},,\\ U_{\mathrm{bend}}\left(\theta ;k_{\theta },r_{\theta },cb\right)=\frac{k_{\theta }}{2}{\left(\theta -\theta_{0}\right)}^{2}\left(1+cb\left(\theta -\theta_{0}\right)\right),,\\ U_{\mathrm{stretch-bend}}\left(r_{ij},r_{jk},\theta ;k_{s-b}{,}_{ijk},k_{s-b}{,}_{kji},r_{ij}{,}_{0},r_{jk}{,}_{0},\theta_{0}\right)=\left(k_{s-b}{,}_{ijk}\left(r_{ij}-r_{ij}{,}_{0}\right)+k_{s-b}{,}_{kji}\left(r_{jk}-r_{jk}{,}_{0}\right)\right)\left(\theta -\theta_{0}\right),,\\ U_{\mathrm{torsion}}\left(\phi ;k_{\phi ,1},k_{\phi ,2},k_{\phi ,3},\phi_{0}\right)=k_{\phi ,1}\left(1+\cos\left(\phi -\phi_{0}\right)\right)+k_{\phi ,2}\left(1-\cos2\left(\phi -\phi_{0}\right)\right)+k_{\phi ,3}\left(1+\cos3\left(\phi -\phi_{0}\right)\right),,\\ U_{\mathrm{Coulomb}}\left(r;q_{i},q_{j},k_{C},\delta \right)=k_{C}\frac{q_{i}q_{j}}{r+\delta },,\\ U_{\mathrm{vdW}}\left(r;\epsilon_{ij},r_{ij}^{*},r_{0}\right)=\epsilon_{ij}\left[{\left(\frac{1.07r_{ij}^{*}}{r_{ij}+0.07r_{ij}^{*}}\right)}^{7}\left(\frac{1.12r_{ij}^{*7}}{r_{ij}^{7}+0.12r_{ij}^{*7}}\right)-2\right], \end{array}$$

where variables are listed before semicolon, while parameters are listed after it.

The Espaloma Class II model was trained on bonded energy terms described in eq 2 which were isolated from total QM molecular energy by means of RDKit^41^ and OpenFF Toolkit^42^ software programs (see Subsection [Sec sec3.2]). Non-bonded parameters for MMFF94 Class II functional forms in *U*_Coulomb_ and *U*_vdW_, as specified in ref 27, were imported from Dompé’s LiGen Virtual Screening software.^43^

## 3 Methods

A recent trend in virtual screening campaigns is to include millions of molecules on multiple targets, leading to extreme-scale virtual screening campaigns.^44,45^ One implication is that the chemical library increases the storage requirements. To mitigate this issue, it is possible to encode the ligand in SMILES and expand them at runtime. In this case, the expansion must have a tight integration with the virtual screening software and sustain its throughput. In this article, we target LiGen-Pre, a software module of LiGen that generates the 3D structure of molecules starting from their string representation. LiGen-Pre is part of the EXSCALATE virtual screening platform.^45^

### 3.1 Ligen-Pre Software Structure

LiGen^43^ is a High-Throughput Virtual Screening (HTVS) pipeline that efficiently screens large-scale drug datasets, filtering out suboptimal candidates to identify a refined subset of small-molecule ligands for subsequent experimental validation in wet laboratories. By leveraging in silico methods, LiGen significantly enhances the efficiency of the drug discovery process, reducing the time and cost. Due to the high storage demands associated with large-scale drug datasets, drug candidates cannot be stored as 3D molecular conformations. Instead, in the initial step of the LiGen workflow, molecules stored as 1D representations (typically SMILES) must be expanded into their appropriate 3D conformations, guided by an optimization algorithm alongside a force field. This preprocessing step is performed by LiGen-Pre, a dedicated module within LiGen.

To build the molecular Potential Energy Surface (PES) using force field parameters predicted by Espaloma, we took advantage of LiGen-Pre.

Figure 1 illustrates the workflow of LiGen-Pre. Initially, SMILES generates the molecule’s initial three-dimensional conformation based solely on its topological information. Subsequently, an optimization algorithm iteratively refines the atomic coordinates to progressively approach a molecular conformer associated with a local energy minimum. In LiGen-Pre, the Limited-Memory Broyden Fletcher Goldfarb-Shanno (LBFGS) algorithm is employed for this purpose. If LBFGS fails to converge appropriately, then the BFGS algorithm is used as a fallback. During the optimization process, the total energy of the molecule is computed by using a functional form provided by the force field. LiGen-Pre employs the MMFF94 force field, a Class II force field that shares the same functional forms included in Espaloma Class II, as described in Section 2.2. This guarantees that force field parameters predicted by Espaloma Class II can be readily used by LiGen to build the molecular PES. Furthermore, the parameters for the MMFF94 functional form are sourced from a precompiled parameter table, enabling rapid lookup based solely on each atom’s type. When valid force field parameters are not found within such tables, step-down procedures, empirical rules, and default parameters are used instead. Such approximations are not necessary when replacing MMFF94 parameters with Espaloma Class II ones, since the model always predicts valid force field parameters for the parsed input molecule thanks to the message passing approach and continuous embedding property.

**Figure 1:**
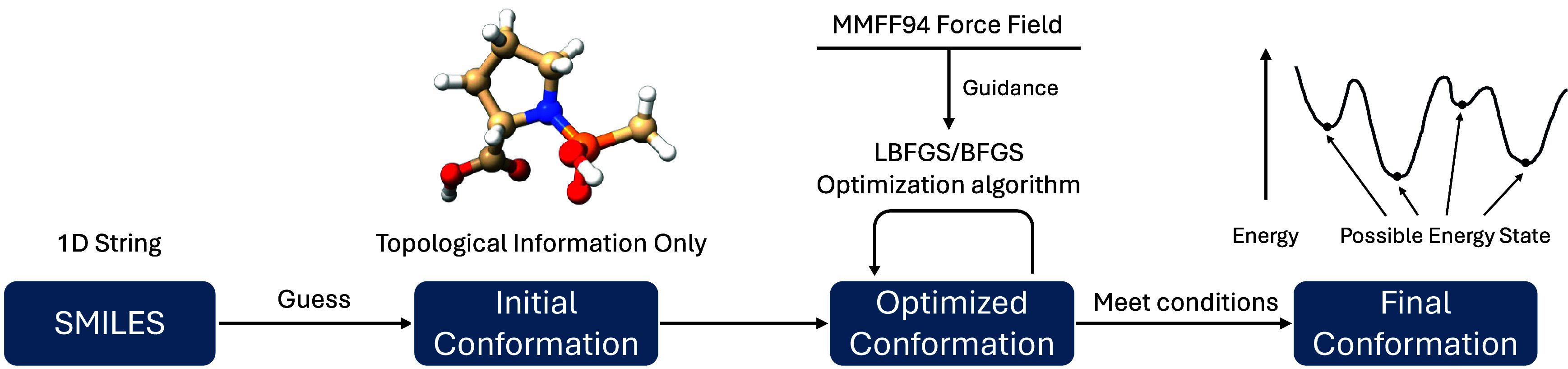
LiGen-Pre workflow.

On the other side, to consistently use Espaloma Class I predicted parameters, we replaced LiGen’s MMFF94 functional forms with those used by Espaloma Class I ones, defined in ref 25 and described in eq 2.

### 3.2 Training, Validation and Test of Espaloma

Espaloma is a Machine Learning Force Field based on a Graph-Neural Network (GNN) architecture. The network takes as input the chemical graph of the molecule and produces in output force field parameters for the molecule at hand according to prespecified functional forms. For each given node in the molecular graph, network input features are atom’s one-hot encoded element, hybridization state, aromaticity, ring membership, and formal charge. The chemical sensing of Espaloma is implicit in the atom embeddings.

We retrained the Espaloma force field following the methodology and code provided by the original authors (https://github.com/choderalab/refit-espaloma). For Espaloma Class I, no modifications were necessary with respect to the original codebase (https://github.com/choderalab/espaloma), as it retains the original parametrization workflow. Related Class I functional forms were integrated in LiGen code^43^ to consistently build molecular PESs using valence parameters predicted by Espaloma model.

On the other hand, to generate a new Espaloma model predicting force field parameters according to Class II functional forms, we constructed new training and validation datasets using the provided code and the following datasets: SPICE-Pubchem, SPICE-DES-Monomers, Gen2-Opt, Gen2-Torsion, and SPICE-Dipeptide. Following the approach used in Espaloma Class I, where non-bonded interactions are subtracted from the total input energy, we estimated non-bonded interaction energy terms for MMFF94 by using RDKit and subtracted them from training energy labels. Since Espaloma does not explicitly model non-bonded energies, this step ensured that the model could be consistently trained on MMFF94 based bonded energy terms. Additionally, to maintain compatibility with MMFF94 functional forms, we converted units accordingly: distances from Bohr to Angstroms and energies from Hartree to kJ/mol.

A key step in our model extension was the replacement of Class I functional forms with the corresponding Class II functional forms, as detailed in Section 2.2. For example, linear molecules are explicitly handled in our MMFF94 based chemical sensing scheme and force field using dedicated atom types and related functional forms, which are not present in the OpenFF 2.0.0 functional forms used in Espaloma 0.3.1. Furthermore, proper and improper torsional terms in Espaloma Class II differ from those in Class I. MMFF94 uses a harmonic function to model improper torsion, also known as “out-of-plane bending”, and enforce planarity of related structures. We ran SMARTS query to determine improper torsions, and we included in Espaloma a new out-of-plane bending energy term to explicitly model improper torsional energy contributions, thus ensuring consistency with LiGen^43^ expecting Class II force field parameters to be integrated within MMFF94 functional forms.

We trained Espaloma Class II for 1000 epochs, using a mean squared error (MSE) loss between the predicted and reference energies, following the same optimization strategy described by Takaba et al.^24^

### 3.3 Valence Parameters Condensation

The first step in using a force field entails assigning proper atom types to molecular moieties according to the chemical sensing model with respect to which the force field was parametrized according to prespecified functional forms. After the molecule has been parsed, force field parameters are assigned to specific atom types and a molecular Potential Energy Surface (PES) depending on molecule’s internal variables is built. Subsequently, the PES is scanned using proper minimization algorithms to determine its minima, which correspond to molecular conformers.

The computational complexity of a force field includes the operations required to assign functional form parameters. Transferable force fields, such as MMFF94,^27^ hinge on straightforward tabular data structures that have negligible access time. These tables can be loaded directly into memory at runtime, facilitating efficient parameter retrieval through simple lookup operations based on the atomic types of molecule’s atoms. In contrast, Machine Learning Force Fields (MLFFs) involve a complex inference process for parameter assignment, usually with python-only bindings. Consequently, MLFFs are treated as external modules in HTVS. For instance, in the case of Espaloma, the external module is an application that expects the molecule structure to be analyzed in the input to compute the predicted parameter set provided in the output.

To quantitatively assess the impact of MLFFs on computational performance, we assessed the performances from transferable force fields to those from MLFFs as detailed in Section 4.1. Due to substantially longer prediction times, MLFFs require 9× more computational time with respect to transferable force fields under identical experimental conditions. This level of performance is impractical for computationally intensive applications, such as High-Throughput Virtual Screening, where efficiency is critical.

To address the degradation in computational performance caused by the MLFFs’ prediction process, we propose a condensation approach that involves tabulating the parameters predicted by the machine learning model. This method systematically organizes the model’s outputs into a structured, readable table that hinges on discrete atom types to map the values of all the potential energy terms. The general concept is illustrated in Figure 2. The left side depicts the inference process of MLFFs, with Espaloma used as an example. In this process, the complete molecular topology is provided as an input, and the machine learning model is used to infer the parameters required for each functional form. The right side illustrates the condensation approach, where the full and computationally intensive inference process is bypassed. Instead, the required parameters are efficiently retrieved through a simple lookup based on the atom types of the corresponding molecular segment.

**Figure 2:**
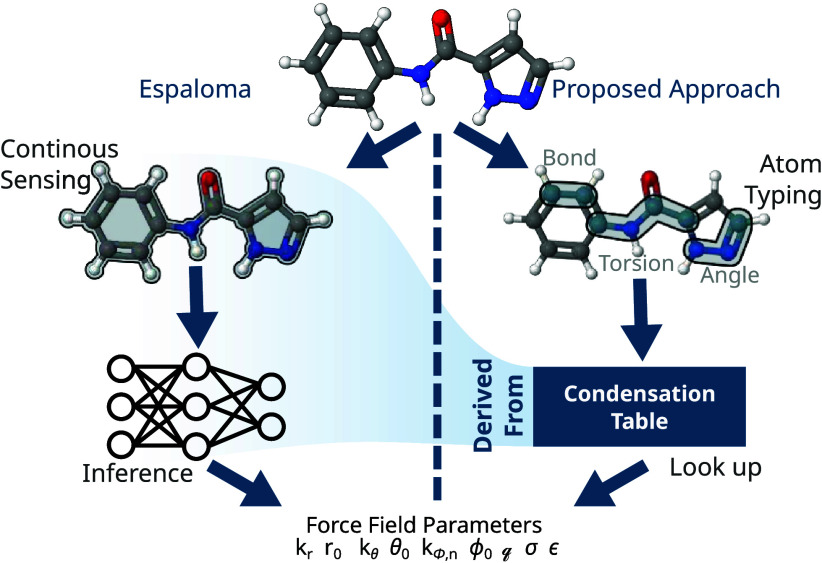
Overview of how the original Espaloma approach (on the left) and the proposed one relying on condensation (on the right) derive force field parameters. Both of them start from the structure of a molecule and predict the same set of force field parameters. The actual value of the parameters is usually different.

The chemical sensing model can be chosen according to the specific context. For this study, we adopted an atom-typing scheme consistent with the transferable force field MMFF94.^27^ This scheme includes 100 atom types, offering sufficient versatility and a comprehensive representation of atomic diversity to meet the requirements of common High-Throughput Virtual Screening applications. The condensation table is also based on atom types.

Worth noting, a discretization is introduced by the assignment of prespecified atom-types in the handling of the molecule’s chemical environment. More precisely, the discretization is introduced in the assignment of force field parameters according to a representative discrete space instead of being continuously predicted according to molecule-specific atom embeddings. However, once a molecule is parsed and atom types assigned, a continuous molecular PES is built using the selected force field parameters (uniquely defined for the molecule at hand) and continuous functional forms. Since the functional forms we use are also differentiable with respect to the molecule’s internal variables, the molecular PES can be minimized to finally retrieve molecular conformers.

Of course, as we will see in 4.2, the assignment of prespecified atom types and related force field parameters gives rise to a slightly less accurate PES than the one that can be obtained using continuous embedding and molecule-specific parameters assignment. However, we stress that the use of the former avoids runtime predictions, which would otherwise render the approach not compatible with High Throughput Virtual Screening speed requirements.

In Figure 3, the *X*-axis represents all prediction results for the reference angle of Espaloma for the MMFF94 atom type combination 1–1–1 (i.e., alkyl carbon, alkyl carbon, alkyl carbon) in the training dataset, while the *Y*-axis shows the count of each statistical bin.

**Figure 3:**
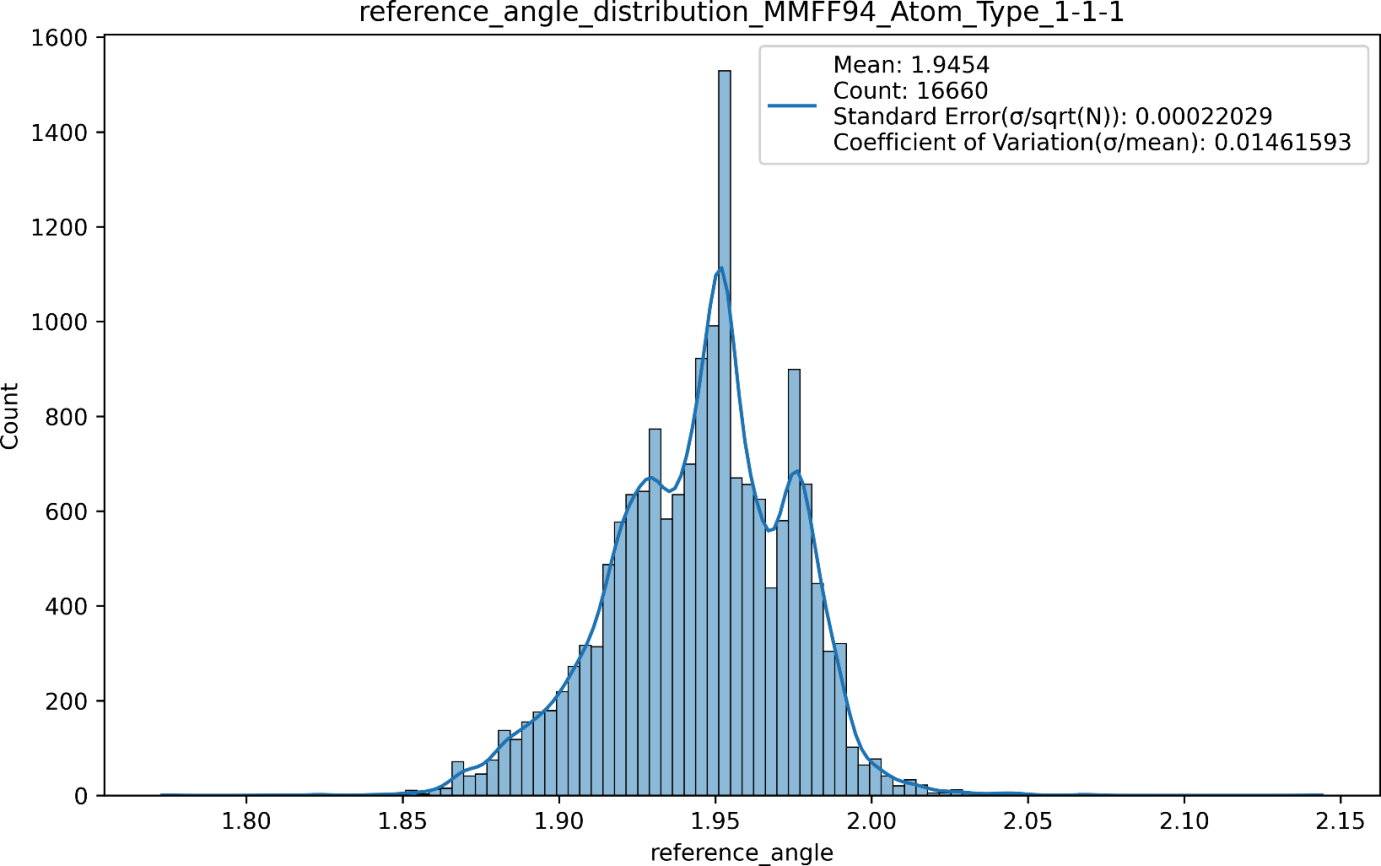
Prediction space of Espaloma reference angles for alkyl carbon.

The prediction space exhibits an approximately normal distribution, spanning a narrow range of values between 1.75 and 2.15. Given this limited variability, a single representative value, such as the median or the mean, can effectively capture the distribution’s central tendency without substantial loss of accuracy. This simplification allows for an approximate yet computationally efficient approach, which is particularly useful when the value is later incorporated into a more complex functional form, as in our case, that remains robust to small variations in force field parameters.

In the current work, we tested three different statistical measures within the condensation workflow as representative values for the central tendency in numerical distributions of force field parameters, namely, the mean, the median, and the mode. For the specific training and test sets used in this work, we found out that distilling the numerical distributions of force field parameters according to the mean value generates a slightly more accurate force field for molecular structure prediction than using the median or the mode ones.

In the benchmarks appearing throughout the rest of this work, the Espaloma condensed force field was generated using the mean as the descriptor for the distribution’s central tendency. Indeed, we provide the reader with the RMSD and TFD performance reached on the OpenFF Industry Benchmark Season 1 v1.1 test set obtained by using the mean value as the central indicator.

The detailed workflow for generating the condensation table is illustrated in Figure 4. Initially, the dataset was collected from the OpenFF QCArchive.^46^ From this archive, we selected five subsets: SPICE-PubChem, SPICE-DES-Monomers, SPICE-Dipeptide, Gen2-Opt, and Gen2-Torsion, comprising a total of 17794 molecular structures. Subsequently, the dataset was preprocessed to extract the molecular conformation files in MOL2 format from the source files. These MOL2 files were then used as input for Espaloma to infer parameters, ultimately generating the metadata for each molecule’s parameters. Notably, the source dataset includes multiple conformers for each molecule. Since Espaloma perceives the chemical environment solely from the molecular topological graph and is independent of specific conformations, any conformer of a given molecule can be used. Additionally, during the experimental process, we observed that certain molecules contained errors, leading to processing failures in Espaloma. If such errors occur on a limited scale, then they can be addressed by excluding the affected molecules. In our case, parameters were successfully generated for 13773 out of the 17794 molecules.

**Figure 4:**
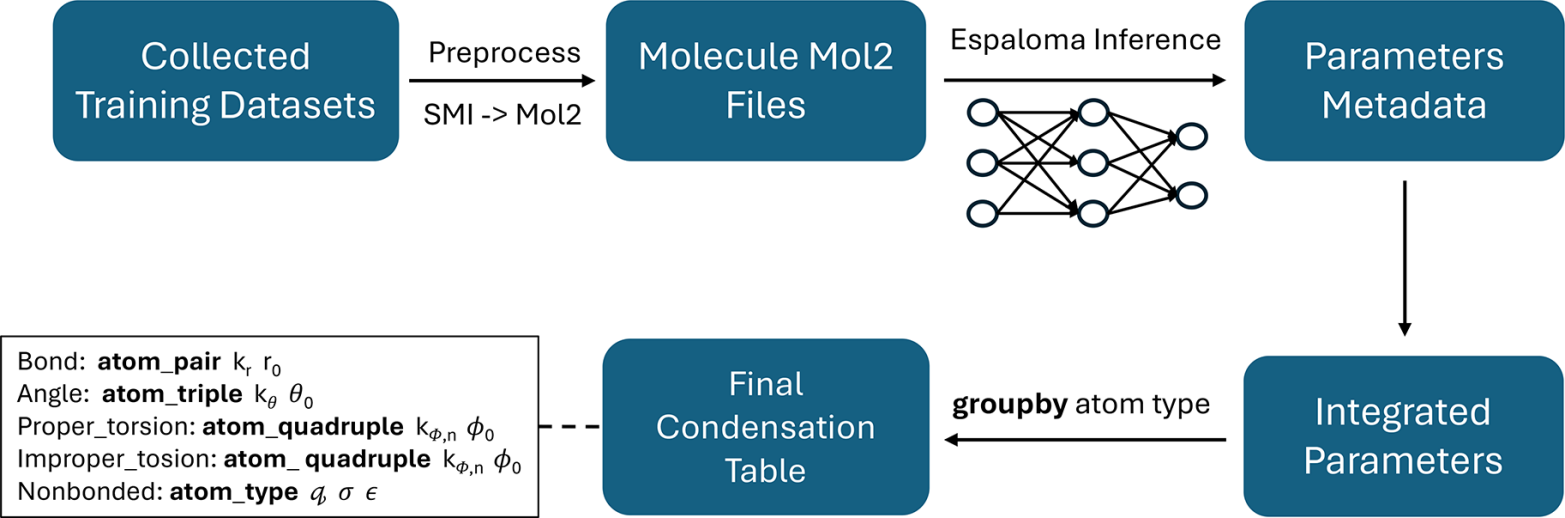
Condensation table generation workflow.

After having collected the metadata for all molecular parameters in the dataset, the latter were integrated based on the categories defined by the force field. For instance, in the Espaloma Class I framework described in Section 2.1, the categories include Bond Stretching, Angle Bending, Proper Torsional Interactions, Improper Torsional Interactions, and Non-Bonded Interactions. Once the data is integrated into these five categories, a group-by operation is applied to specific atomic types. For example, force field parameters related to atom-pair couples such as Alkyl Carbon–Alkyl Carbon in the Bond-Stretching category are grouped accordingly. Finally, the mean value is computed and designated as the representative value for each force field parameter in the Final Condensation Table.

Notably, the Condensation Table Generation Workflow presented in Figure 4 is designed to potentially incorporate all parameters specified by the force field. However, as reported by the authors of Espaloma in,^25^ the machine learning model is used to infer bonded parameters only while non-bonded ones are borrowed from an existing transferable force field. As a consequence, the condensation table in this study focuses on bond parameters only.

In addition, the process we used to generate the tables containing the condensed force field parameters was designed to cover the majority of the chemical space that can be assessed in High-Throughput Virtual Screening campaigns. However, when in PES modeling we need to use the value of a force field parameter that is not present in the condensation table, we disregard the contribution, which means that the final energy of the molecule will not include the involved interaction. Section 4.2 evaluates the proposed approach that uses this strategy.

## 4 Results and Discussion

This section presents experimental evaluations of the proposed condensation scheme for the force field parameters predicted by the Espaloma machine learning model. The experiments are structured into two parts: one assessing the computational performance of the force fields and the other evaluating their predictive accuracy.

Since for High-Throughput Virtual Screening (HTVS) applications we’re mostly interested in molecular structure optimization tasks, we benchmarked widely used transferable biomolecular force fields on RMSD and TFD performances. We leave for future studies more computationally demanding assessments such as delta delta Energy (ddE) evaluation as defined in ref 47 and force field reliability in Molecular Dynamics (MD) simulations for protein–ligand Absolute Binding Free Energy (ABFE) calculations.

Although such tasks can already be performed accurately using established ML approaches like Neural Network Potentials (NNPs, e.g.,^11,13^ ) or ML-corrected semi-empirical (ML-SE) methods (e.g., PM6-ML,^48^ DelFTa^49^), their computational performances are still at least 1–2 orders of magnitude lower than those reached by optimization algorithms based on Class I force fields. Therefore, NNPs and ML-SE methods are not used in large scale HTVS campaigns and may only be considered in later stages of the screening workflow (e.g., false positives ratio reduction).

### 4.1 Force Fields Computational Performance Comparison

To quantitatively assess the computational performances of various force Fields, we integrated them into the molecular expansion pipeline in LiGen, as detailed in Section 3.1. The evaluation process began by partitioning the test dataset into batches of 100 molecules each, followed by generating their initial conformations. Subsequently, the pipeline, embedded with the force fields under evaluation, was used to compute the 3D Conformations of all molecules across all batches, while the execution time for each batch was recorded. For the experiments, calculations were performed by using parallel computing on an AMD EPYC 7282 processor with 16 cores. We allocated all the computation resources to the Espaloma application to perform the typing, which hinges on PyTorch to parallelize the batch computation. The LiGen executable is serial.

The experimental results are presented in Figure 5, where we evaluate the computational performance of four force fields: the Espaloma Class I, the force field obtained using the Condensation approach on the parameters predicted by Espaloma Class I, the transferable Class II force field MMFF94 and the Espaloma Class II. In the figure, the *X*-axis represents the execution time per batch, while the *Y*-axis denotes the density of each bin in the distribution. Approaches relying on indirect chemical sensing to assign force field parameters, i.e., MMFF94 and Condensed Espaloma, are significantly faster than Espaloma. We were expecting this result since explicit atom typing only involves parsing of the immediate neighbors of the atom at hand. On the other side, Espaloma uses transformers to extract node features based on the whole molecular graph and a fully connected neural network to predict force field parameters, which introduces a significant computational effort. Moreover, the atom typing and table lookup implementations are written in C while the Espaloma implementation is still written in Python, due to Python-only dependencies.

**Figure 5:**
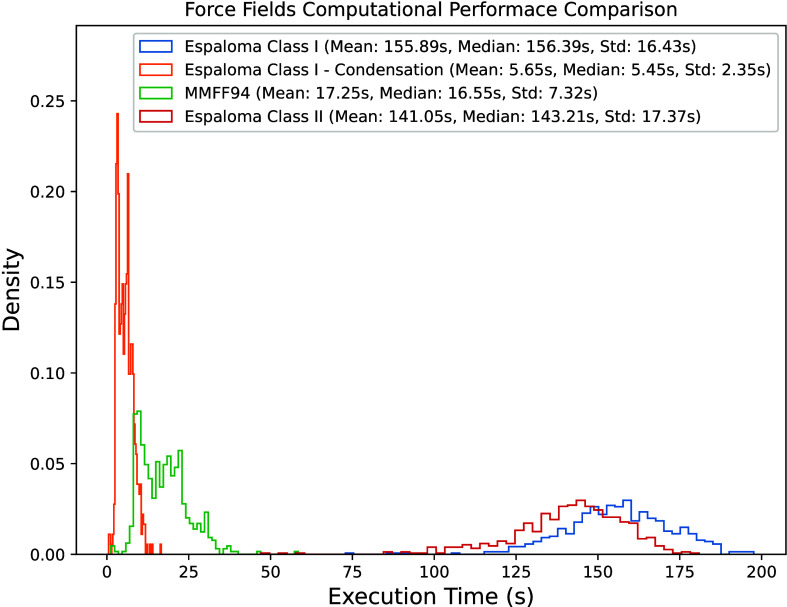
Force fields computational performance comparison.

### 4.2 Force Fields Predictive Accuracy Comparison

To assess force fields’ predictive accuracy, we followed the evaluation method proposed by D’Amore et al.,^47^ together with their published dataset. This approach involves using force fields to recompute conformers of a given molecule that were previously optimized with high precision quantum mechanical methods, the QM-optimized conformation serving as ground truth.

In this framework, we investigated whether our condensation scheme for force field parameters predicted by Espaloma preserves the reliability of the original model in quantitatively reproducing quantum chemical molecular structures. In fact, such an ability is of capital importance when biomolecular force fields are used for modeling molecular docking phenomena or for scoring promising ligands in Virtual Screening (VS) campaigns using either fast Scoring Functions (SF) or more computationally demanding methods such as Absolute Binding Free Energy (ABFE) calculations from Molecular Dynamics (MD) simulations.

Therefore, we assessed the accuracy of molecular conformers obtained using our condensation scheme on numerical distributions predicted by the Espaloma 0.3.1 model. In the benchmark we also included the Espaloma model itself and several other well established transferable biomolecular force fields such as MMFF94, OpenFF 2.0.0, Gaff 2.11, Opls4 cst, and Opls4 def. QM-optimized gas-phase molecular geometries from the standardized industrial benchmark dataset were used as reference structures for computing RMSD and TFD values. More in detail, the referenced dataset is the OpenFF Industry Benchmark Season 1 v1.11, a collection of drug-like molecules chosen by Open Force Field Consortium industry partners and indicative of their current interests in Computer Aided Drug Design domain.^47^ Such reference structures are calculated at the DFT level of theory using dispersion corrected hybrid functional (B3LYP-D3BJ) and double-ζ valence polarized (DZVP) basis set that have been demonstrated to be highly accurate for calculating drug-like molecules ground state equilibrium geometries.”

Theoretically, the most accurate force field should produce a conformation with minimal RMSD and TFD values with respect to the QM-optimized structure or with respect to an alternative molecular conformer corresponding to a nearby local minimum.

Worth noting, this assessment approach defines as reference structures multiple molecular conformers associated with PES’ local minima instead of the single molecular conformer associated with PES global minimum, allowing one to decouple the assessment of force field accuracy for molecular structure optimization tasks from the assessment of the search algorithm performances in identifying the global minimum, whose handling is usually not mandatory in standard HTVS campaigns.

In order to visually illustrate the energy variations involved in the evaluation process, Figure 6 presents a simulated 2D projection of the high-dimensional Potential Energy Surface (PES). The black curve represents a hypothetical “true” energy landscape, where each black point corresponds to a local minimum conformation. The orange curve represents a possible energy landscape associated with the force field under evaluation. Ideally, the force field’s energy curve should align perfectly with the black curve, ensuring that the MM optimized conformer coincides with the molecular conformer whose energy is represented by one of the four black points. In most cases, the MM optimized conformer only deviates slightly from its original structure and stays within the same energy basin. However, in certain instances, such as the scenario depicted by the orange curve, the new conformation may transition into a different local basin. In such cases, the molecular conformer associated with the newly reached local minimum is selected as the new reference for computing RMSD and TFD values. This is because the deviation between the final MM optimized conformer and the molecular conformer associated with the newly reached local minimum is smaller than the corresponding deviation with respect to the molecular conformer associated with the original local minimum.

**Figure 6:**
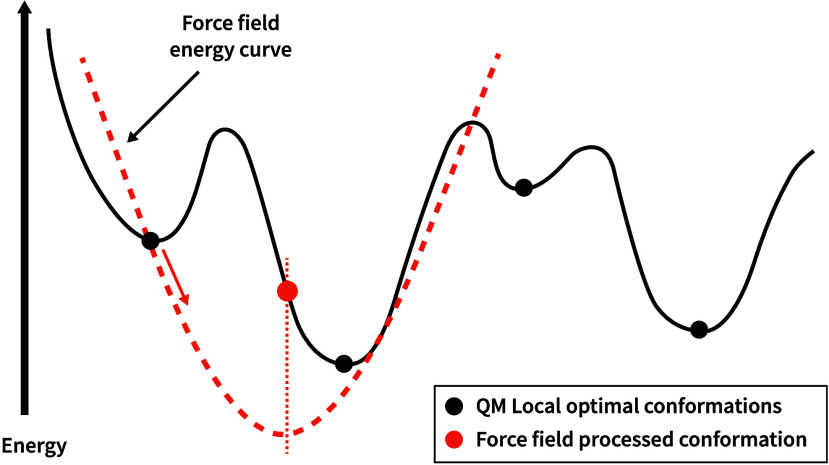
Schematic representation of force fields energy curve.

The specialized evaluation workflow is illustrated in Figure 7. We first use the force field under evaluation to recompute the optimized conformation of each molecule in the dataset. The newly generated conformer is then compared to the original QM one and other local minima provided in the dataset. To quantify these differences, we compute the root-mean-square deviation (RMSD) and Torsion Fingerprint Deviation (TFD)^50^ values. Given two aligned molecular conformations, RMSD quantifies the average positional displacement of corresponding atoms between the structures, while TFD captures the difference in their dihedral angles, reflecting variations in the torsional state.

**Figure 7:**
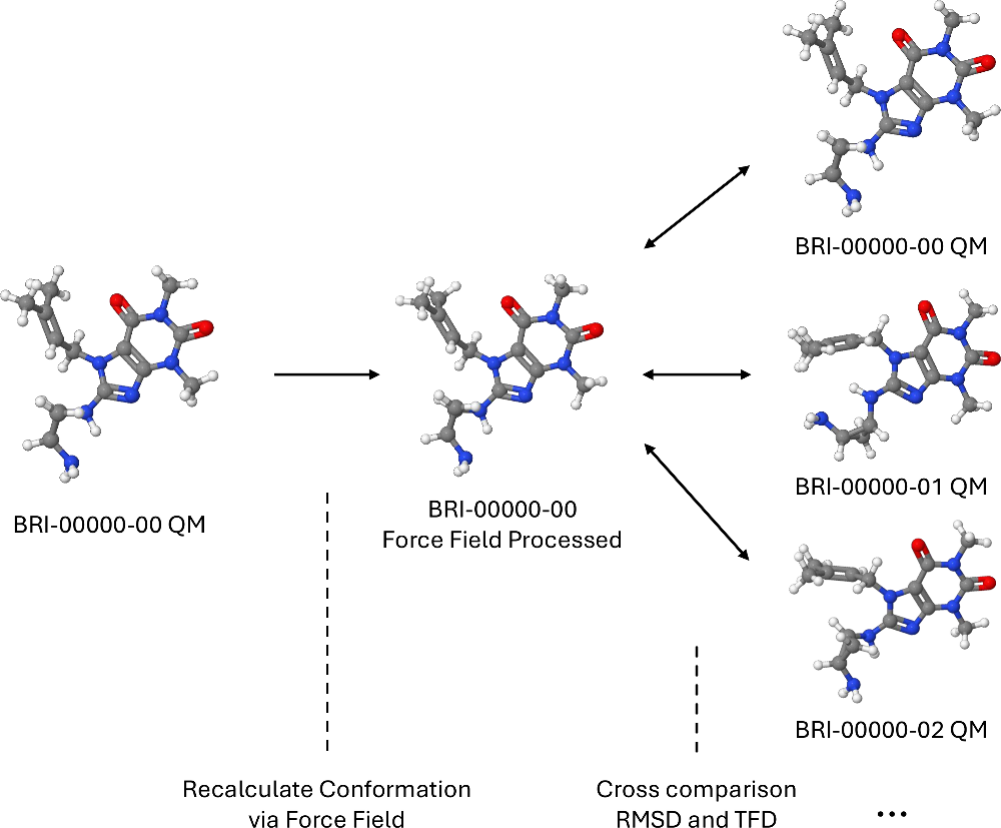
Force fields predictive accuracy evaluation workflow.

The results are presented in Figure 8, which depicts the distributions of the RMSD and TFD values on the left and right, respectively. The *X*-axis represents the binning of specific values, while the *Y*-axis denotes the cumulative density of each bin. Under this configuration, a higher cumulative density within smaller RMSD/TFD intervals indicates greater predictive accuracy of the force field under evaluation. The figure compares four Force Fields: Espaloma Class I, the force field obtained from Espaloma Class I using the condensation approach, the transferable biomolecular Force Field MMFF94, and Espaloma Class II. As noted in Section 4, the condensation approach is applied to distill only to bonded parameters, whereas non-bonded ones are borrowed from OpenFF 2.0.0.

**Figure 8:**
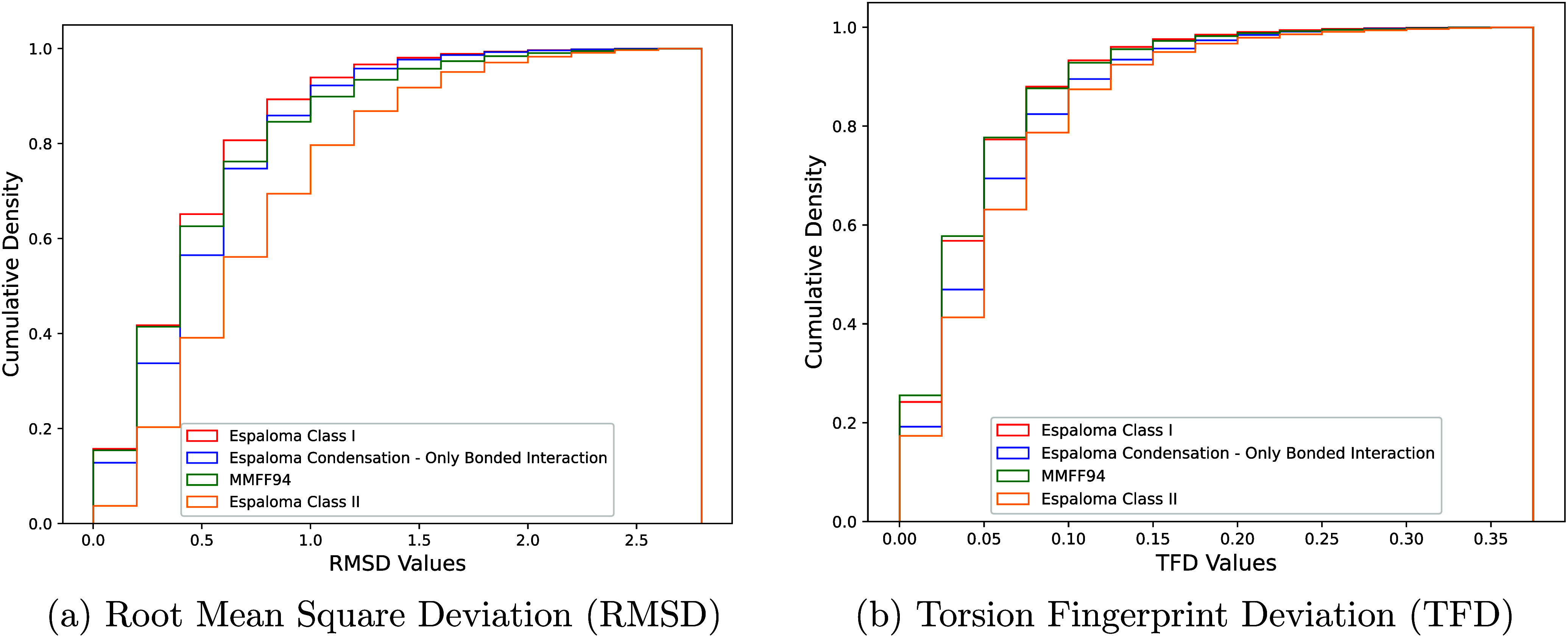
Force fields predictive accuracy comparison in terms of RMSD (a) and TFD (b).

Overall, Espaloma Class I demonstrates a slight advantage over MMFF94 but given that MMFF94 is a Class II force field, the superiority of Espaloma Class I becomes more remarkable. In comparison to the force field generated by the original Espaloma model, its condensed version exhibits a slight decrease in prediction accuracy. However, this trade-off is justifiable considering the substantial improvement in computational efficiency, as illustrated in Figure 5. Specifically, the molecular structure generation process using the latter force field achieves a 30-fold speedup with respect to the corresponding process using the former one. In parallel, the decline in prediction accuracy remains within a maximum of 0.1 in terms of density. These findings suggest that the condensation approach is a promising strategy for balancing computational efficiency and predictive accuracy, making it more practical than the original Espaloma approach for real-world HTVS applications, where efficiency is a key factor. Moreover, compared to MMFF94, the condensation approach not only improves computational efficiency but also benefits from a data-driven, flexible, and continuously updated condensation table, as discussed in Section 4. Unlike transferable force fields, this process operates without requiring expert intervention, offering significant advantages in adaptability and scalability.

Finally, we benchmarked on the OpenFF Industry Benchmark Season 1 v1.1 dataset the statistically validated force field against the one provided by the original Espaloma model, the one provided by the Espaloma model equipped with Class II functional forms and other well-known transferable biomolecular force fields, as shown in Table 1.

**Table 1:** Machine Learning Force Fields and Transferable Force Fields RMSD and TFD Performances on OpenFF Industry Benchmark Season 1 v1.1

	Espaloma Class I	Espaloma Class II	Condensed Espaloma	MMFF94	Openff 2.0.0	Gaff 2.11	Opls4 cst	Opls4 def
RMSD mean value	0.542	0.835	0.607	0.598	0.54	0.65	0.328	0.362
TFD mean value	0.055	0.071	0.065	0.055	0.046	0.061	0.036	0.039
RMSD median value	0.464	0.722	0.543	0.474	0.386	0.502	0.23	0.262
TFD median value	0.044	0.059	0.053	0.043	0.034	0.045	0.026	0.029
No. of conformations	58681	58262	58527	58945	58487	58514	58610	58610

Espaloma 0.3.1 is in line with that from OpenFF 2.0.0. The slight loss of accuracy with respect to OpenFF 2.0.0, a consequence of having condensed numerical distributions of force field parameters predicted by Espaloma 0.3.1, allows for using the condensation approach and offline computing instead of being compelled to rely upon runtime ML predictions.

Furthermore, our condensation scheme offers several advantages over transferable force fields, namely:

- Fast retraining upon upgrade/introduction of new functional forms;
- Active learning schemes can be used to efficiently select new training instances reducing the risk of over- and underfitting on both existing and new training datasets, a balance that is much harder to attain for a transferable force field;
- Opportunity to develop a consistent protein–ligand force field by using the same condensation scheme;
- Opportunity to develop force field parameters with respect to new atom types or even chemical sensing models without changing the condensation workflow.

Finally, although Class II functional forms give rise to more accurate Potential Energy Surface (PES) and thus should in principle provide more accurate molecular structures, the force field generated using the Espaloma model equipped with Class II functional forms shows worse performances than the one generated using the Espaloma model equipped with Class I ones.

This mainly happens because predicting force field parameters with a machine learning model requires minimizing a loss function whose parameters can differ by orders of magnitude (e.g., equilibrium distances and angles vs related elastic constants). Such a combination turns out to give rise to an ill-conditioned problem, i.e., nearly parallel response vectors in system equations, and although one can choose directly optimize force field parameters, such an approach would give rise to significant difficulties in training. This is a well-known problem also in transferable biomolecular force field development. For example, the robustness issue in optimizing molecular mechanics force field parameters was also observed during the parametrization of the CHARMM General Force Field (CGenFF) where different contributions (like those from vibrational frequencies) are mixed.^51^ Such an ill-conditioned problem is faced both when dealing with Class I functional forms and when dealing with Class II functional forms. However, when fitting harmonic, uncoupled (i.e., Class I) functional forms, the almost underdeterminedness (or, more precisely, the ill-conditionedness) of the system can be resolved by taking advantage of a linear decomposition strategy recently proposed by K. Vanommeslaeghe et al.^52^

As described in ref 25, Appendix B.1, Training and inference, eqs 32–34, stage III of the Espaloma model equipped with Class I functional forms takes advantage of the referenced decomposition strategy to predict individual terms of the linear sum, which are then used to recover optimized force field parameters. Unfortunately, this decomposition strategy is effective only with harmonic, uncoupled loss functions (as originated from Class I functional forms), while for anharmonic, coupled loss functions (from Class II functional forms), a similar decomposition method does not exist yet.

This is why, although equipped with more accurate functional forms, the Espaloma model equipped with Class II functional forms brings about a loss function (squared energy error as a function of original force field parameters), which is much more difficult to minimize due to the unresolved large difference in gradient magnitudes between force field parameters to be simultaneously optimized.

## 5 Future Work

Current version of Espaloma training set does not cover many of the bond stretching, angle bending, and proper and improper torsions parameters encompassed by Class I functional forms and the MMFF94 chemical sensing model. In this framework, Query-by-Committee Active Learning approaches are well suited to expand the model Applicability Domain with efficient use of computational resources.

Finally, to improve model’s accuracy in describing Protein–Ligand non-covalent interactions, we plan to take advantage of Espaloma modularity to include tailored intermolecular functional forms to explicitly handle directional H-bonding, π-π stacking, and ion-dipole interactions.

## 6 Conclusions

In this work, we assessed whether it is possible to take advantage of the accuracy of predicted MLFF parameters while retaining computational speed compatible with High-Throughput Virtual Screening (HTVS) campaigns. We used the Espaloma 0.3.1 model for massively predicting force field parameters according to predefined Class I functional forms.

To begin, we assessed whether the Espaloma Class II model provided more accurate molecular structures than its Class I counterpart. Therefore, we equipped the Espaloma model with Class II functional forms, integrated the resulting MLFF in Dompé’s LiGen Virtual Screening software, and assessed it on the OpenFF Industry Benchmark Season 1 v1.1 against several well-known biomolecular transferable force fields. We then took advantage of the Espaloma model and MMFF94’s atom types to generate numerical distributions for each valence force field parameter found in five subsets of the original Espaloma training set, namely: SPICE-PubChem, SPICE-DES-Monomers, SPICE-Dipeptide, Gen2-Opt, and Gen2-Torsion datasets. Finally, we condensed each numerical distribution related to a specific force field parameter by extracting its mean value. This procedure allowed us to capture in a statistical sense the effect on a given force field parameter brought about by different chemical environments in molecules sharing the parameter at hand.

We then assessed on the OpenFF Industry Benchmark Season 1 v1.1 dataset the accuracy of molecular geometries generated using force field parameters provided by the Condensed Espaloma workflow. Compared to the original Espaloma model, we only found a minor decrease in Root Mean Squared and Torsional Fingerprint Deviations while observing a 30× increase in computational efficiency. Finally, to give more context, we assessed the force field generated using the Condensation workflow against several well-established biomolecular transferable ones.

Our method is designed to provide statistically validated force field parameters that can be transferred to model different molecular environments. We believe our approach will pave the way for future developments of Machine Learning Force Fields for High Throughput Virtual Screening applications.

## Data Availability

All data and software used to generate the results in the paper are publicly available. Raw data and Python code related to the proposed condensation approach are available at https://github.com/elvispolimi/forcefield-condensation. The original ESPALOMA code and training procedure for the model is also made available from the original authors, respectively, at https://github.com/choderalab/espaloma and https://github.com/choderalab/refit-espaloma. The OpenFF Industry Benchmark Season 1 v1.1 dataset used to assess the molecule geometries generated is publicly available at https://zenodo.org/records/15801401.
